# Contrast-enhanced ultrasound: a new tool for imaging the superficial lymphatic vessels of the upper limb

**DOI:** 10.1186/s41747-022-00270-4

**Published:** 2022-04-12

**Authors:** Olli Lahtinen, Ritva Vanninen, Suvi Rautiainen

**Affiliations:** 1 Diagnostic Imaging Centre, Department of Clinical Radiology, Kuopio University Hospital, Kuopio, Finland; 2 Institute of Clinical Medicine, Unit of Radiology, University of Eastern Finland, Kuopio, Finland

**Keywords:** Contrast media, Injections (intradermal), Lymphatic vessels, Sonazoid, Ultrasonography

## Abstract

**Background:**

Despite the new lymphatic imaging methods, there is still a need for a straightforward method of detecting lymphatic abnormalities. Our goal was to investigate the feasibility of applying a contrast enhanced ultrasound (CEUS) procedure as a new approach for visualising the superficial lymphatic vessels of the upper limb.

**Methods:**

Thirty healthy volunteers were examined with CEUS after bilateral intradermal injection of Sonazoid® contrast agent in distal antebrachium. We registered factors affecting intradermal injections, imaging of the superficial lymphatic vessels and the enhancement time of contrast agent reaching the levels of elbow and axilla.

**Results:**

CEUS imaging of superficial lymphatic vessels was successful in 59 of 60 upper limbs (98.3%). Median [interquartile ranges] enhancement times of contrast agent to reach the elbow (right 18 s [11–25], left 15 s [12–25]) and axilla (right 77 s [33–118], left 66 s [42–115]) were equally fast. Successful intradermal injections were found to result in two types of contrast enhancement (strong or moderate), while the enhancement time depended on the type of the successful injection. No major differences in enhancement times were observed related to sex, body mass index, age, or side of the arm.

**Conclusions:**

The superficial lymphatic pathways of the upper limb can be visualised with CEUS imaging. Since enhancement time is dependent on the success of intradermal injections, one must pay attention to the injection technique. Further studies are needed to evaluate the method in patients with lymphatic function disorders such as breast cancer therapy related lymphoedema.

## Key points

- Superficial lymphatic vessels of the upper limbs can be visualised with contrast-enhanced ultrasound.
- Successful intradermal Sonazoid® injections were followed by fast lymphatic drainage.
- Contrast enhancement in the axillary area was detected with a median time of 75 s after the injection.

## Background

Despite the important role of the lymphatic system in human health and diseases, this system is still poorly understood. After the pioneering work of Mascagni [1] and Sappey [2] using a mercury injection method, recent studies with radiopaque lead oxide mixtures in cadavers have led to a re-evaluation of the anatomical details of the lymphatic pathways [3, 4]. Similar to the situation with the vascular system, the anatomy of the lymphatic system consists of a complex network of small vessels. The lymphatic network is composed of the initial lymphatics or lymphatic capillaries, precollectors and collecting vessels [5–7]. The lymphatic capillaries are blind-ended vessels containing a single layer of endothelial cells. The diameter of the initial lymphatic vessels typically o ranges from 20 to 70 μm. Unlike the lymphatic capillaries, precollectors and collecting vessels contain valves to prevent a backflow of lymph fluid. The walls of collecting vessels have smooth muscle cells and collagen fibres that maintain efficient lymph drainage [7]. However, unlike the vascular system the lymph drainage through lymphatic vessels is not propelled by pulsatile blood circulation.

Traditionally, lymphoscintigraphy has been the method of choice for diagnosing lymphatic disorders such as lymphoedema [8]. Recently, new lymphatic imaging methods have developed such as magnetic resonance (MR) lymphangiography and indocyanine green (ICG) fluorescence imaging [9–11]. However, all these imaging techniques are either time consuming, need to expose the patient to radioactive tracer or are not cost effective on a large scale. Therefore, there is a need for a quick, easy and readily available tool with which to assess common lymphatic abnormalities such as secondary lymphoedema after lymphadenectomies of breast cancer surgery [12–14].

Ultrasound (US) might possess some of these advantages since the contrast agent used for contrast-enhanced ultrasound (CEUS) is a water-based solution like ICG which is known to travel faster in lymphatics than is the case with non-water based solutions. Ultrasound has been applied in imaging of the lymphatic system as is in the case of evaluation of lymph nodes (LN) [15]. In breast cancer-related lymphoedema, US can assess the response to physical therapy [16]. Recently, ultra-high frequency US has been applied as an intraoperative planning tool in lymphatic microsurgery [17]. Additionally, the sentinel lymph nodes (SLNs) and related afferent lymphatic vessels have been successfully visualised with CEUS in breast and vulvar cancers [18–22].

CEUS is one of the new US approaches using microbubbles such as those of perflubutane (Sonazoid®, GE Healthcare, Oslo, Norway) used in this study. Perflubutane is a relatively new, second generation US contrast agent with some additional characteristics over the more commonly used sulfur hexafluoride (Sonovue®, Bracco, Milan, Italy) such as its affinity for reticuloendothelial cells [23, 24]. Like sulfur hexafluoride, it is well tolerated and has no major contraindications or severe known adverse effects when administered by intravenous or subdermal injections [25, 26].

In the present study, our aim was to investigate the feasibility of applying CEUS to achieve a fast visualisation of the superficial lymphatic vessels of the upper limbs in healthy volunteers. In addition, we registered possible confounding factors related to the intradermal injection technique to be applied with the microbubble contrast agent.

## Methods

### Patients and study design

Approvals from our institutional review board, local ethics committee and Finnish Medicines Agency (FIMEA) were obtained for this prospective single-centre study and all of the participants provided written informed consent.

Thirty healthy volunteers were recruited to the study. Both upper limbs were independently evaluated in each subject. Exclusion criteria were (1) egg protein allergy; (2) pregnancy; (3) breastfeeding; (4) prior axillary lymphadenectomy. To allow us to evaluate age dependence, the volunteers were further divided into two groups: a younger group of persons less than 40 years of age and an older group equal or greater than 40 years of age. When studying the possible effect of body mass index (BMI), the volunteers were divided into two groups: subjects with BMI < 25 and subjects with BMI > 25.

### Upper limb US and CEUS

The US procedures were performed by a single radiologist with 6 years of experience with CEUS. A Logiq E9^TM^ US-device (General Electric Healthcare, Chicago, USA) with a broad spectrum ML6-15 (6–15 MHz) linear array transducer. Participants were examined in supine position relaxed with no muscle stress. The applied CEUS procedure was performed similar to US studies for the identification of SLNs [18, 20].

### Microbubble contrast agent injection

**Fig. 1:**
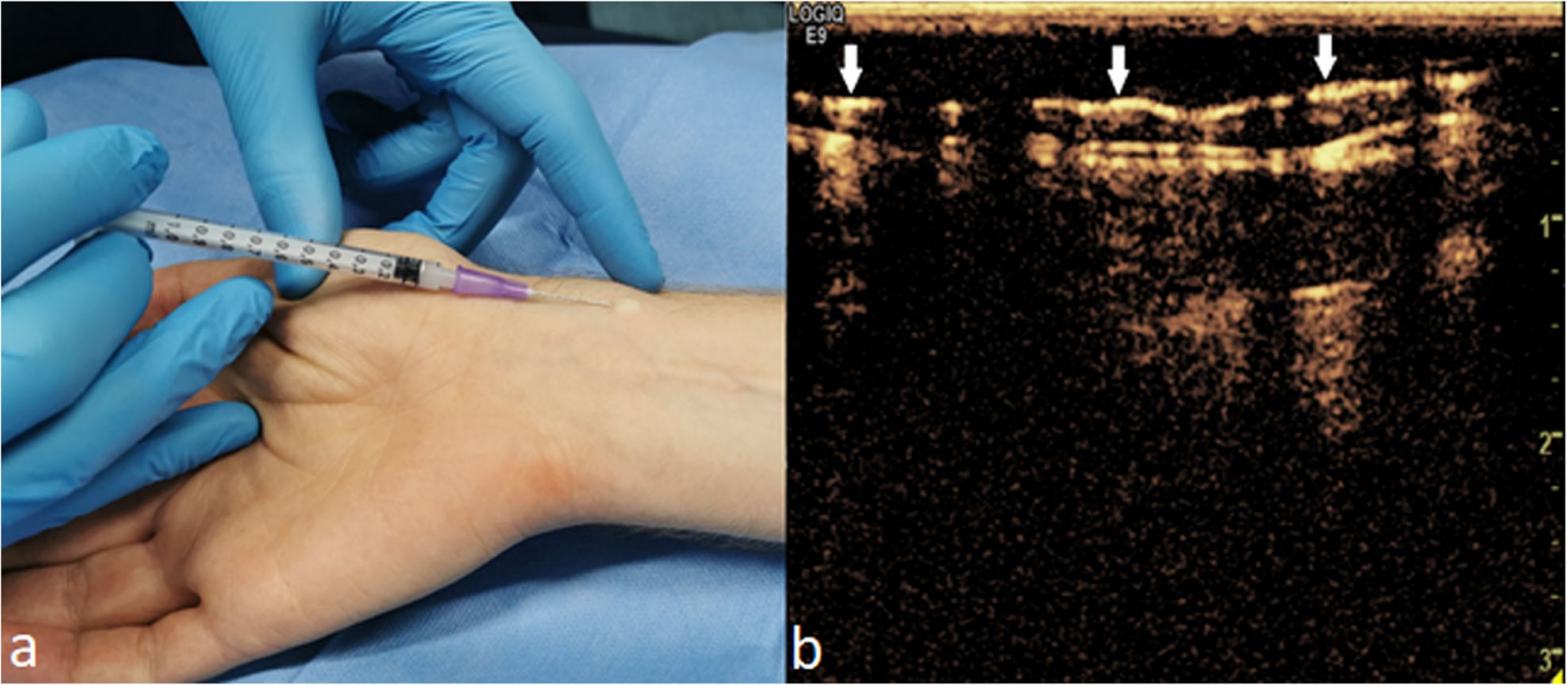
**a** Injection site in the distal antebrachium and intradermal injection technique. A small blister-like spot beneath the skin in addition to resistance to injection are probable indicators of a successful injection. Contrast agent (Sonazoid®, white arrows) can be seen in the superficial lymphatic vessel in **b**

**Fig. 2:**
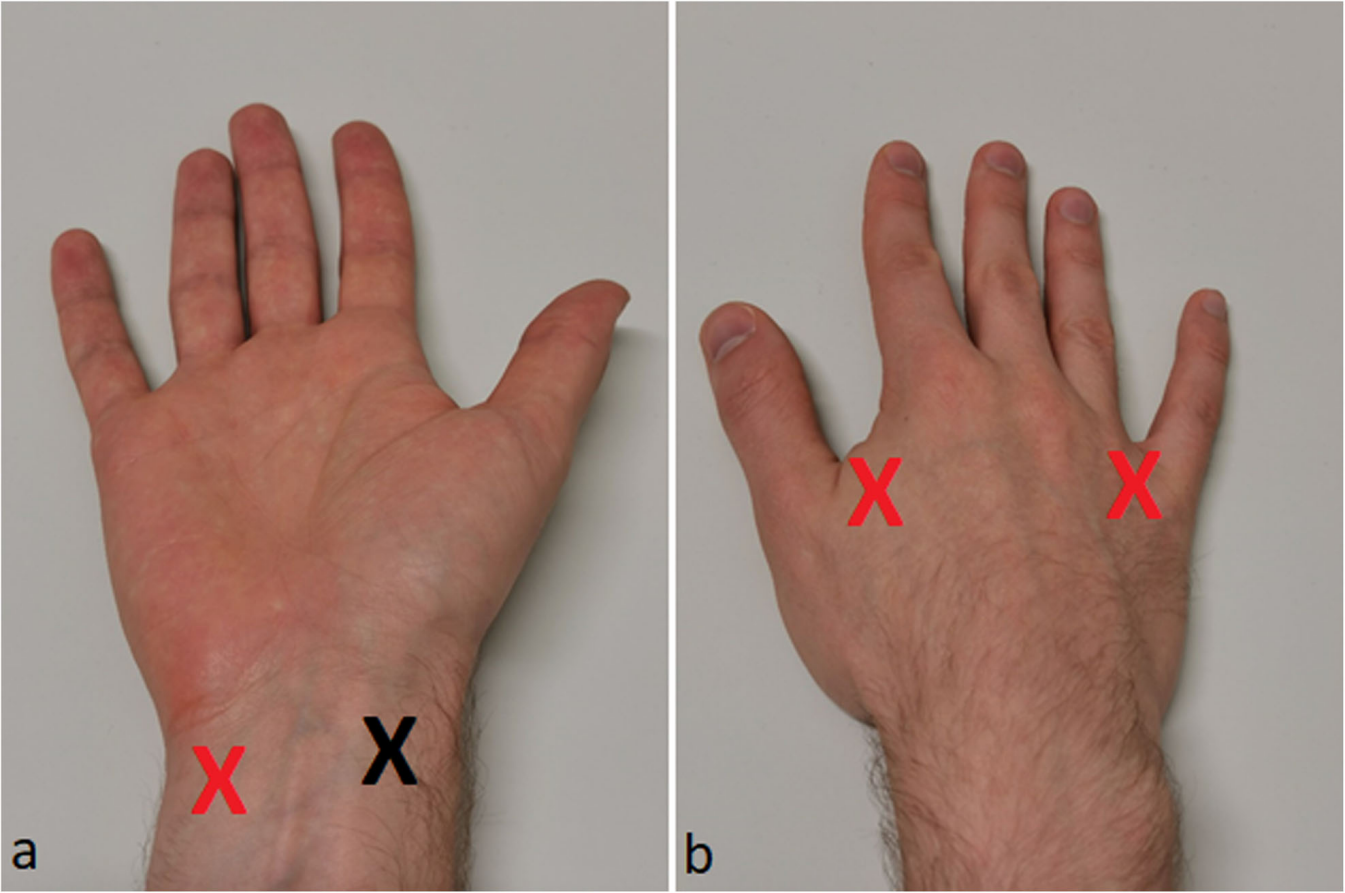
The black X in **a** marks the primary injection site in the distal antebrachium. Additional injection sites are marked with a red X in **a** and **b**

We hypothesised that the required enhancement times for the evaluation of the whole upper limb would be longer compared to those required in SLN studies. Therefore, the contrast agent Sonazoid® was selected for the study as potentially being more durable compared to the more commonly used Sonovue®. The contrast agent powder, Sonazoid® was mixed with 2.0 mL of sterile saline producing a microbubble solution with a mean particle diameter of 2.6 μm. The Sonazoid® particles were injected intradermally with a 1.0-mL syringe and a 24-gauge needle laterally on the volar side of the distal antebrachium (Fig. 1). The injection was made with a low angle, typically less than 5° from the skin. The tip of the needle was positioned so that it could be seen through the thin layer of epidermis. Injection was made as a fast bolus. However, the injection rate depended on the resistance from the tissues. The timer was started after the whole volume was injected. The injected volume of the contrast agent solution was 0.2-mL for the whole study population. If an enhancing lymphatic vessel was not detected after injection or only minimal enhancement was seen near the injection site in the CEUS image, the injection site was gently massaged for approximately 10 s. The injection was repeated up to three times at a nearby skin site within 1 cm from the first injection site if no additional enhancement was seen after the massage. In addition, three other injection sites (Fig. 2) similar to those used by Suami et al. [11] were tested in each upper limb. Injected volumes of 0.1, 0.2, and 0.3 mL were tested in five volunteers after a successful injection to the primary site. Additional injections were made when no residual enhancement was seen from the previous injections. Continuous flash was used before the next injection to help to destroy the microbubbles. Thus, the additional test volumes in the five volunteers were used to test: (1) the feasibility with a lower dose; and (2) whether a stronger enhancement type would follow with the larger dose. Besides feasibility assessment, no detailed evaluation of the different enhancement patterns or times from other injection sites was performed.

**Fig. 3:**
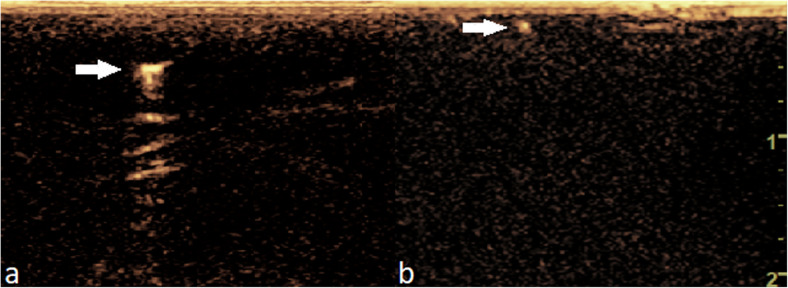
The CEUS image in **a** correlates with a type A (strong enhancement) in the superficial lymphatic vessel, whereas the CEUS image in **b** corresponds to type B (moderate enhancement), which is clearly seen from the background but is clearly more faint than type A. White arrows mark the enhancing superficial lymphatic vessels

The flow of contrast agent was followed using a standard low (< 0.10) mechanical index, as recommended by the manufacturer of Sonazoid®. The CEUS program allows the user to distinguish the echoes emitted by the microbubbles from tissue background and optimise the visualisation of the contrast agent in the US image. The enhancement time from contrast agent injection to the detection of the contrast agent in antecubital fossa and axilla was registered and major lymphatic pathways and the number of enhanced lymphatic vessels were documented. The strength of the enhancement was divided into two categories by the observer, depending on the visual appearance of the contrast enhancement. Category A corresponded to strong enhancement in the superficial lymphatic vessel while category B referred to moderate enhancement (Fig. 3).

### Follow-up

Participants were followed up for any possible acute onset of adverse effects for one hour after the administration of the contrast agent. In addition, the participants were told to contact the main investigator should any late onset symptoms develop within 72 h after the contrast agent injection.

### Statistical analysis

Variables are reported as median and interquartile range. Statistical analysis was performed with SPSS (Version 26.0, SPSS, Chicago, IL, USA). The Mann-Whitney *U* test was used to calculate statistical significance between the different variables (dichotomised age, sex, BMI, strength of the enhancement). Wilcoxon-related samples test was used to calculate inter-side differences between right and left upper limbs of the appearance time of the contrast agent in antecubital fossa and axilla. Values of *p* lower than 0.05 were considered significant.

## Results

Thirty healthy volunteers (16 males and 14 females, aged 41 ± 11.2 years, mean ± standard deviation, range 27–69 years) were recruited between October 2020 and January 2021. Both upper limbs were evaluated in each subject. The BMI was 25.8 ± 4.8 (mean ± standard deviation, range 19.2–39.3). The right arm was dominant in 29 persons.

**Fig. 4:**
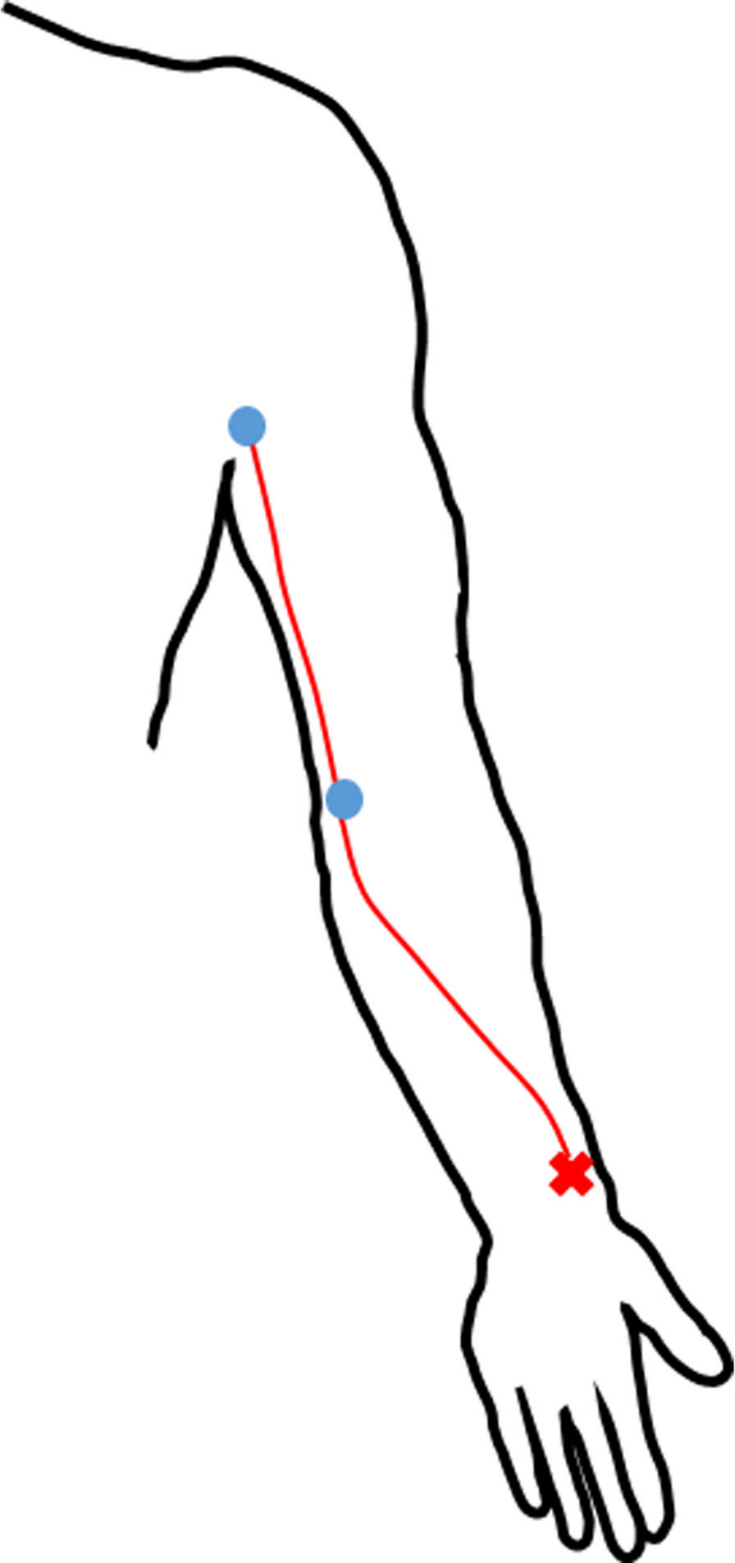
The red X marks the injection site of the contrast agent. Blue dots demonstrate the location of the enhancing lymph nodes in the antecubital fossa and axilla. The red line illustrates roughly the enhancing superficial lymphatic route seen in all of the successful injections

Intradermal contrast agent injections were successful in 59/60 (98.3%) of the upper limbs evaluated. A mean of 3.1 injections were needed to visualise the lymphatic collector vessels from upper arms combined. A clear indicator of a failed injection was the lack of resistance during the administration of contrast agent. The number of enhanced lymphatic collectors varied between 1 and 4 with a mean of 1.5 vessels per upper limb that were located in parallel. In all volunteers, the contrast agent followed the same lymphatic pathway (Fig. 4).

**Fig. 5:**
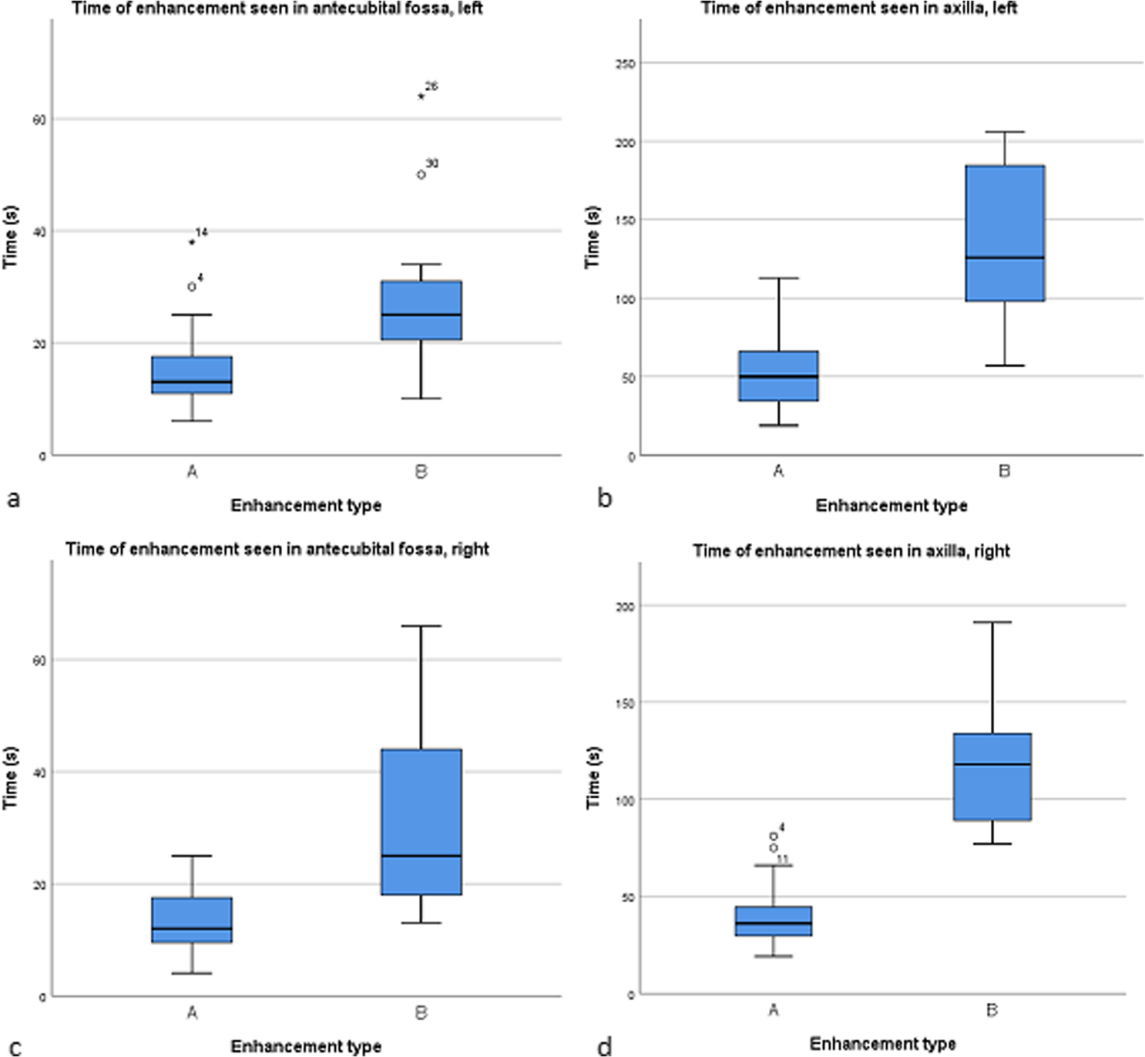
Enhancement times in antecubital fossa (**a**, **c**) and axilla (**b**, **d**) displayed variance according to the two US enhancement categories (type A = strong, type B = moderate) in the right and left arms

The enhancement time of the contrast agent depended on the success of intradermal injections. Typically, a successful intradermal injection produced a blister-like spot with a diameter of about 5 mm. None or only minimal visualisation of the lymphatics was registered if the injection of contrast agent had occurred subcutaneously. Successful intradermal injections resulted into two types of contrast enhancement. In type A (*n* = 34), a high concentration enhancement and a fast enhancement time of contrast agent in lymphatics were observed. Type B (*n* = 25) enhancements demonstrated less noticeable visualisation of the lymphatics with a longer enhancement time in antecubital fossa and axillar area (Fig. 5). In some injections resulting in type B enhancements, the contrast agent was found to have spread into a larger area beneath the skin surface compared to the typical intradermal injections producing a blister-like spot. Thus, type A enhancement pattern could be characterised as being associated with a successful intradermal injection, and a type B enhancement pattern as related to a partially successful intradermal injection. Table 1 summarises the enhancement times of the contrast agent.

**Table 1:** Enhancement times in the antecubital fossa and axilla after intradermal injections

Assessment site	Enhancement time (s) Median (interquartile range)
Right antecubital fossa	18 (11–25)
Left antecubital fossa	15 (12–25)
Right axilla	77 (33–118)
Left axilla	66 (42–115)

There was no statistically significant difference in median enhancement times to reach right antecubital fossa (18 s) and left antecubital fossa (15 s) (*p* = 0.863) or axilla (77 s and 66 s, respectively) (*p*= 0.581). BMI or younger *versus* older age of volunteers had no significant effect on enhancement times. A slight difference was found between genders in the enhancement time in the left antecubital fossa, where females had 28% faster mean enhancement time compared to males (females 18 s *versus* males 23 s, *p* = 0.029). More detailed information is presented in Table 2.

**Table 2:** Effect of different parameters on the enhancement time after successful intradermal injections

			Enhancement time (s) Median (interquartile range)			
Parameter		Number	Antecubital fossa		Axilla	
			Right	Left	Right	Left
Sex	Males	16	15 (10–31)	20 (13–25)	57 (31–120)	98 (52–133)
	Females	14	19 (12–25)	12 (9–21)	81 (42–116)	49 (31–96)
	*p*		0.455	0.029	0.583	0.070
Age (years)	< 40	18	18 (11–34)	19 (11–29)	77 (33–123)	92 (49–147)
	> 40	12	18 (11–25)	15 (12–24)	77 (32–109)	50 (37–95)
	*p*		0.929	0.484	0.825	0.086
Body mass index	< 25	16	19 (13–33)	17 (11–23)	80 (40–123)	69 (39–133)
	> 25	14	14 (11–25)	14 (12–35)	57 (28–99)	63 (44–112)
	*p*		0.256	0.546	0.111	0.884
		Right+ left				
Enhancement type	A	15 + 19 = 34	12 (9–20)	13 (10–19)	36 (29–49)	50 (32–70)
	B	14 + 11 = 25	25 (18–46)	25 (20–34)	118 (88–134)	126 (95–191)
	*p*		0.001	0.007	0.000	0.000

Enhancement type A indicates a successful intradermal injection with strong visual enhancement, whereas type B refers to a partially successful injection with moderate enhancement. Enhancement times in the table represent median enhancement times at the antecubital fossa and axilla

Additional test volumes were used in five volunteers. These demonstrated that in addition to a standard test dose of 0.2 mL, lymphatic vessels could also be visualised with doses of 0.1 mL and 0.3 mL. Furthermore, injections of contrast agent into one additional volar site and into two sites in dorsal aspects on back of the hand were also feasible to visualise the superficial lymphatic routes. The lymph drainage from these additional sites followed the same pathway as with the main injection site on the volar aspect of the distal antebrachium. The alternative lymphatic pathways passing on the lateral aspect of the upper arm following the cephalic vein and connecting straight to the supraclavicular lymph nodes were not visualised in this study. In two subjects, an enhancing lymph node following the main pathway was found in the medial aspect of the elbow (Fig. 4).

No adverse effects were reported related to the intradermal injections or CEUS microbubble contrast agent.

## Discussion

The present study demonstrates that the CEUS method can be used to visualise the anatomy and function of the superficial lymphatics in the upper limbs, offering a feasible radiological tool for the assessment of diseases and conditions affecting the normal lymphatic function such as in patients suffering from problems after breast cancer therapy. Visualisation of lymphatic pathways agrees with previous studies using microbubble contrast agent imaging for the sentinel lymph nodes and animal studies with cutaneous melanoma [18, 20, 22, 27]. The anatomical information on the lymphatic pathways is in agreement with findings from traditional lymphoscintigraphy and MR lymphangiography [8, 28].

### CEUS microbubble technology

Previously, studies with CEUS have been mainly limited to the imaging of the SLNs in breast cancer and vulvar cancer following intradermal injection of contrast agent [22, 18–20]. To the best of our knowledge, the current study is the first to explore the potential of CEUS as a tool for mapping superficial lymphatics in humans. Although several other means for visualising and diagnosing possible lymphatic problems such as ICG and MR lymphangiography are nowadays available [9–11], there is a lack of a quick and cost-effective screening tool.

This study demonstrated in healthy volunteers that the CEUS procedure can be technically successful in 98.3% of upper limbs for visualising the pathways of the superficial lymphatics. The method proved to be quick since contrast agent enhancement in the axillar area was detected with a median time of 75 s after injection. These enhancement times are much faster than those necessary with ICG, which are about 30 min in the antecubital fossa [29]. The early enhancement time is evidently due to the small particle size of the microbubbles and the water-based solution of contrast agent. Due to the fast enhancement times, the contrast agent was seen in the superficial lymphatic vessels for a few minutes after the initial enhancement. Thus, these results could probably be achieved with the more commonly used contrast agent Sonovue® since it shares same kind of qualities as the Sonazoid® and the lifespan of the microbubbles exceeds the enhancement times shown in this study. Furthermore, it has been previously successfully used in the SLN studies to visualise lymphatic vessels [18–20, 30].

### Factors related to intradermal injections

The intradermal injection technique is a procedure with a learning curve. In our study, a mean of 3.1 injections were needed in order to visualise the lymphatic collector vessels of both upper limbs. Successful intradermal injections were followed by fast lymphatic drainage and good visualisation of the contrast agent in lymphatic vessels (Table 2). However, successful injections could also result in slight-to-moderate visualisation of the lymphatics and longer enhancement times in elbow and axillar area. In some of the slower type B enhancements, the contrast agent was observed to be spreading into a larger area beneath the skin surface whereas more typically, intradermal injections produced a blister-like spot with a diameter of about 5 mm. Thus, the type B enhancements might be related to injections partly into dermis and partly into the upper subcutis and these injections could thus be defined as partially successful. In contrast, an indicator of a failed injection was the lack of resistance during the administration of contrast agent. If no enhancement was detected in the lymphatic vessels, it was evident that the injection had been totally subcutaneous.

In both type A (strong) and type B (moderate) enhancement patterns, the route of contrast agent was found to be identical and corresponded to the major pathways detailed in previous cadaver and ICG studies [7, 11]. Even the partially successful injections could thus also provide valuable information on the anatomy and function of the lymphatic drainage. The enhancement time results were registered from the first successful injection regardless of its enhancement type, leading to a relatively large variation in the enhancement times in our relatively small study population.

In our study, the alternative lymphatic pathways passing on the lateral aspect of the upper arm following the cephalic vein and connecting straight to supraclavicular lymph nodes were not visualised. Injections from secondary peripheral injection sites (Fig. 2), similar to those used by Suami et al. [11], only produced findings that the ipsilateral axilla drainage pathway was the major pathway of contrast agent; results are in agreement with those obtained with ICG fluorescence lymphography.

### Initial findings in healthy volunteers

By dividing our group of volunteers according to gender, BMI, or into subjects young or older ages, we attempted to assess possible individual factors affecting the enhancement time of the contrast agent. If one considers gender-related differences, females displayed a 28% faster mean enhancement time than males in left antecubital fossa (18 s *versus* 23 s) with a slight statistically significant association. However, no clear reason for this phenomenon was identified and it might be affected by variables not included in the study such as arm length. Participants lay on the examination table and no muscle stress was induced voluntary that could affect the result. As massaging the injection site in the SLN studies to expedite the lymphatic flow [18, 20], the muscle contractions increase the lymph flow in skeletal muscle [6] and involuntary upper limb movement could potentially result in faster enhancement time. Otherwise no statistical differences were detected.

This microbubble contrast agent method proved to be feasible in mapping the superficial lymphatics of the upper limbs in healthy volunteers. The method needs to be further tested in patients with lymphatic disorders such as breast cancer-related lymphoedema associated with an abnormal distribution of lymph fluid into dermis. Studies planned for the future should help us to fully understand differences in CEUS lymphatic imaging between the upper limbs of healthy volunteers and patients with lymphoedema. Moreover, since lymphatic anatomy after breast cancer surgery may totally differ from normal lymphatic anatomy [31], benefits, and limitations of the CEUS for imaging the routes of abnormal lymphatic drainage in sites with dermal backflow need to be investigated. Furthermore, lymphatic CEUS studies in patients with breast cancer-related lymphoedema are therefore warranted since dermal backflow is considered as the most reliable indicator for lymphoedema [32] and US devices are widely available in healthcare units.

## Study limitations

The present study has several limitations. Enhancement times were registered quantitatively, but the division into two different enhancement types was done subjectively as whether the intradermal injection was successful, leading to a type A (strong) enhancement or partially successful leading to a relatively large number of type B (moderate) enhancements. Classification into these two types was subjective although the difference could be seen on the live display. The use of time intensity curve analysis, not available for this study, could provide more quantitative results of these types in the future.

Another limitation is the lack of multiple observers, also adding subjectivity to our results. However, a similar injection technique has been used in SLN studies and has shown to be reproducible both in patients with breast cancer and vulvar cancer [20, 21]. Since enhancement time is dependent on the success of intradermal injections, the injection technique demands special care to avoid unsuccessful injection. A reproducible injection technique is crucial if one is to differentiate real lymphatic disorders from injection-induced effects. Further studies investigating the CEUS technique in the longer range of the whole upper limb are warranted to fully clarify the differences between normal and abnormal lymphatic anatomy.

## Conclusions

From this preliminary study, we conclude that the CEUS method following intradermal injections was able to identify the superficial lymphatic pathways in the upper arms of healthy volunteers. CEUS represents a potential minimally invasive tool with which to assess the kinetics of lymph fluid and allow the imaging of abnormal lymphatic anatomy.

## Data Availability

The datasets used and/or analysed during the current study are available from the corresponding author on reasonable request.

## Abbreviations

BMI: Body mass index
CEUS: Contrast-enhanced ultrasound
ICG: Indocyanine green
MR: Magnetic resonance
SLN: Sentinel lymph node
US: Ultrasound
